# Supplementary material for: Impact of KIF4A on Cancer Stem Cells and EMT in Lung Cancer and Glioma
Source: Cancers (Basel). 2023 Nov 22;15(23):5523. doi: 10.3390/cancers15235523 (PMC10705730; doi:10.3390/cancers15235523)
Supplement: Supplementary file 1 [file cancers-15-05523-s001.zip › cancers-2682242-SI.pdf]

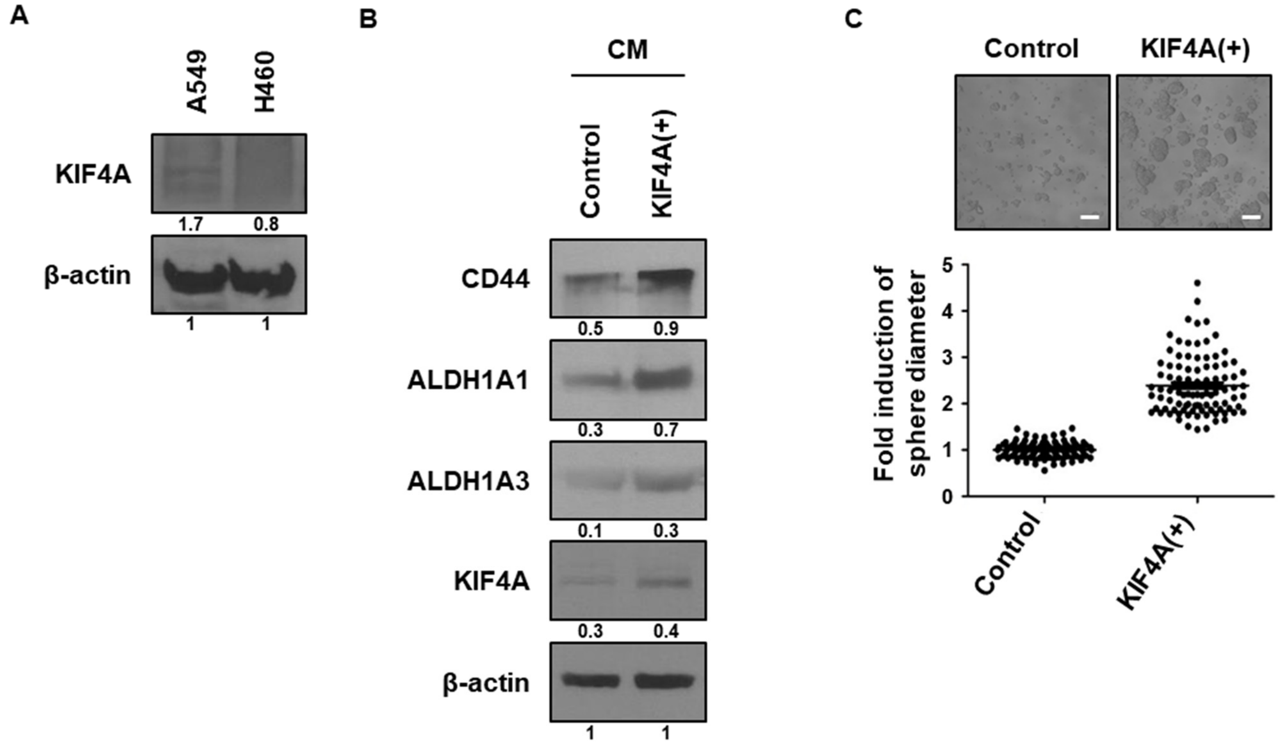

Figure S1. Investigation of KIF4A-mediated effects on lung cancer stem cells by using H460 lung cancer cells. (A) Western blot analysis of the expression of KIF4A in A549 and H460 lung cancer cells. (B) Western blot analysis of the CSC marker proteins by H460 cells which overexpressing KIF4A by overexpression vector. (C) A sphere forming assay was performed by treating H460 cells with KIF4A overexpression vector. Data are presented as the mean ± standard deviation of three each independent experiments.

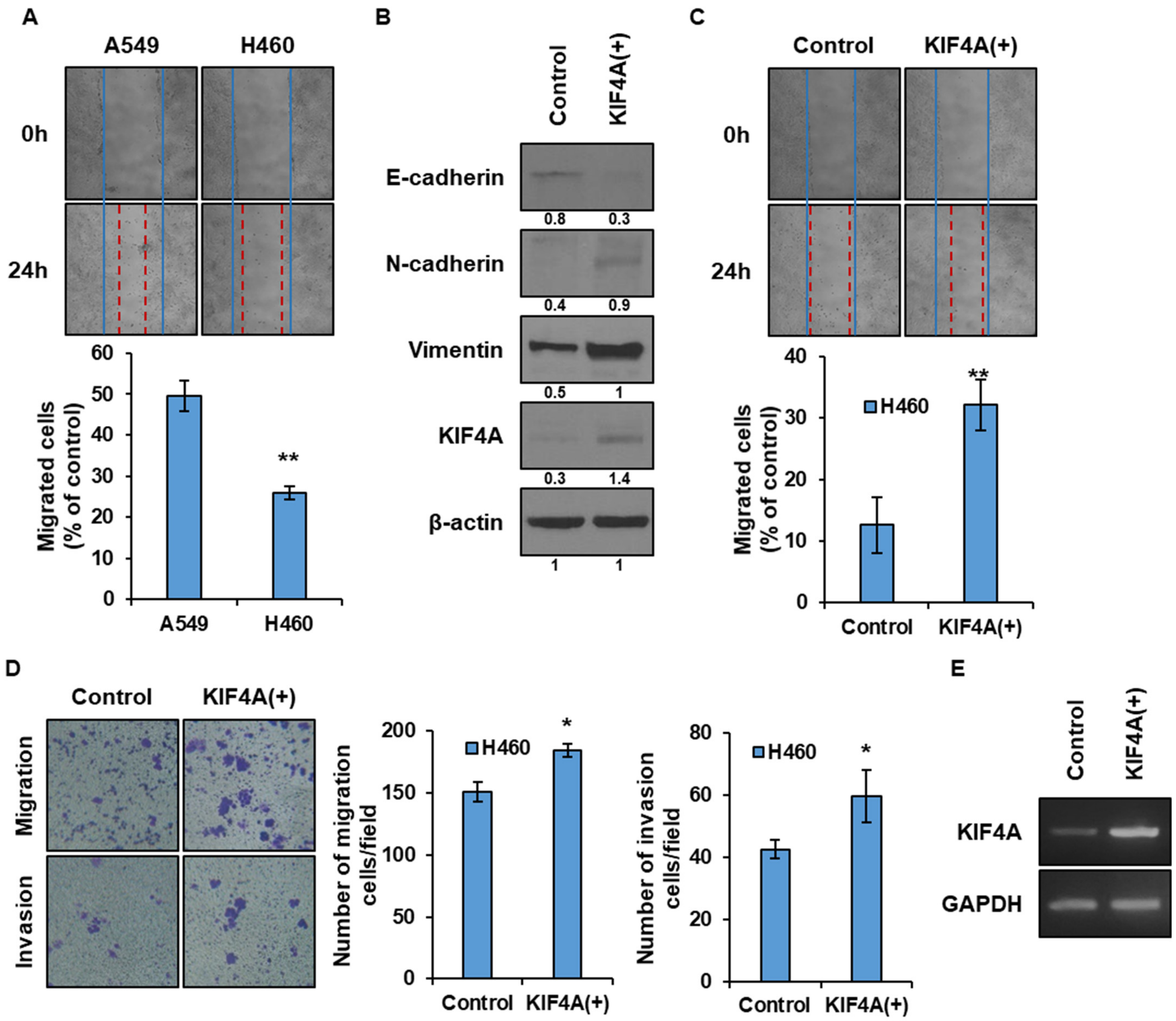

Figure S2. Observation the ability of EMT in H460 cells by overexpressing KIF4A. (A) Comparison of metastatic specificity in A549 and H460 lung cancer cells by Wound healing assay. (B) Western blot analysis of the EMT marker proteins by H460 cells which overexpressing KIF4A by overexpression vector. (C) Comparison of metastatic specificity in control H460 cells and KIF4A overexpressing H460 cells by Wound healing assay. (D) Invasion and migration assay of H460 cells overexpressing KIF4A. (E) Comparison of the expression of KIF4A in control H460 cells and KIF4A overexpressing H460 cells by qPCR. Data are presented as the mean ± standard deviation of three each independent experiments. Scale bar, 50μm. * $p \leq 0.05$, ** $p \leq 0.005$.

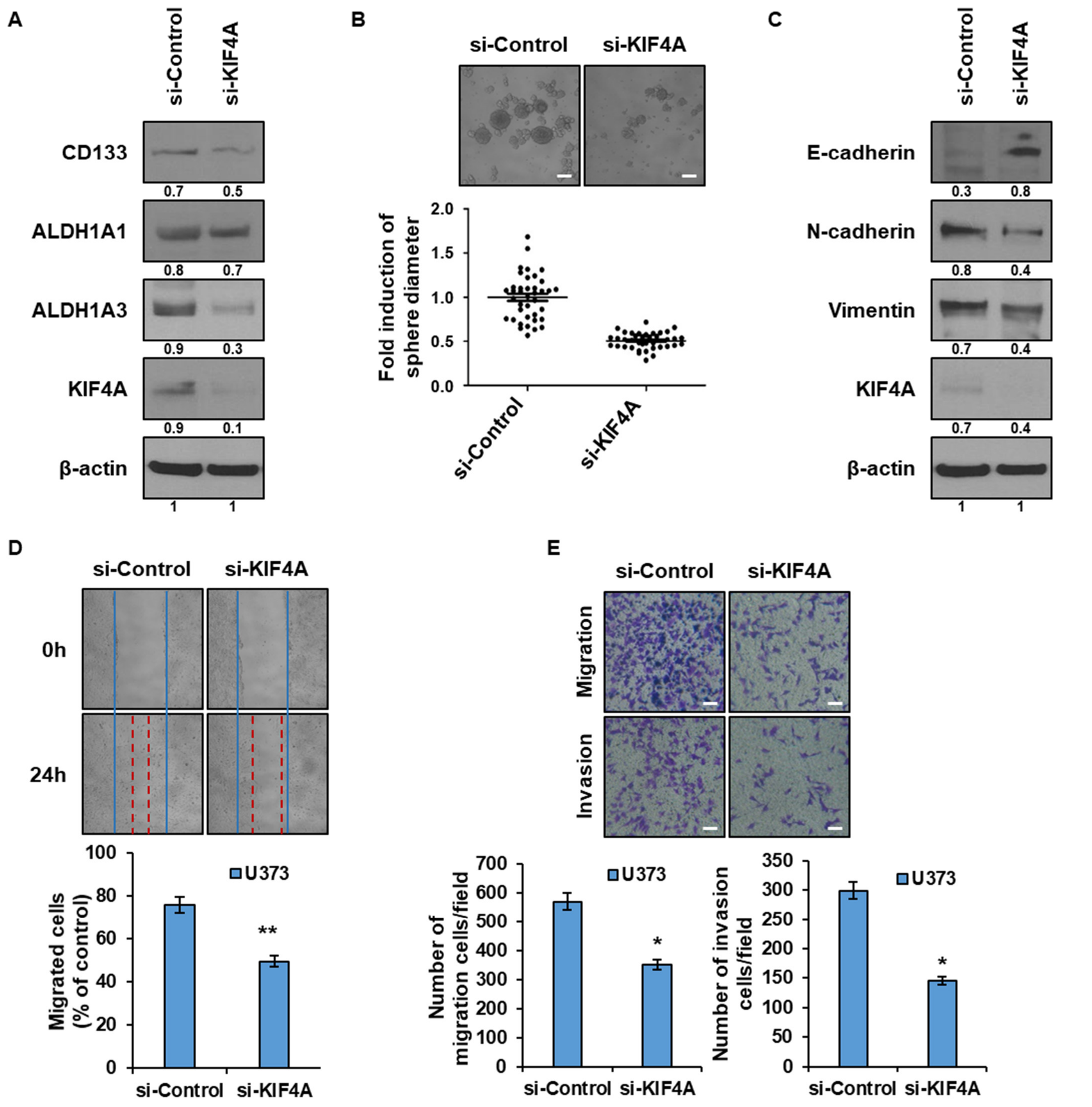

Figure S3. Observation the ability of CSC and EMT in U373 glioma cells. (A) Western blot analysis of the expression levels of CSC marker proteins (CD133, ALDH1A1, ALDH1A3) were compared and analyzed. (B) U373 cells were transfected with si-RNA and then cultured on sphere forming media to verify the sphere forming ability of U373 cells. (C) After inhibiting the expression of KIF4A in U373 cells, the expression level of EMT marker proteins (E-cadherin, N-cadherin, Vimentin) were analyzed by Western blot analysis. (D) Comparison of metastatic specificity in control si-RNA treated U373 cells by Wound healing assay. (E) Invasion and migration assay of KIF4A inhibited U373 cells. Data are presented as the mean ± standard deviation of three each independent experiments. Scale bar, 50µm. * $p \leq 0.05$, ** $p \leq 0.005$.

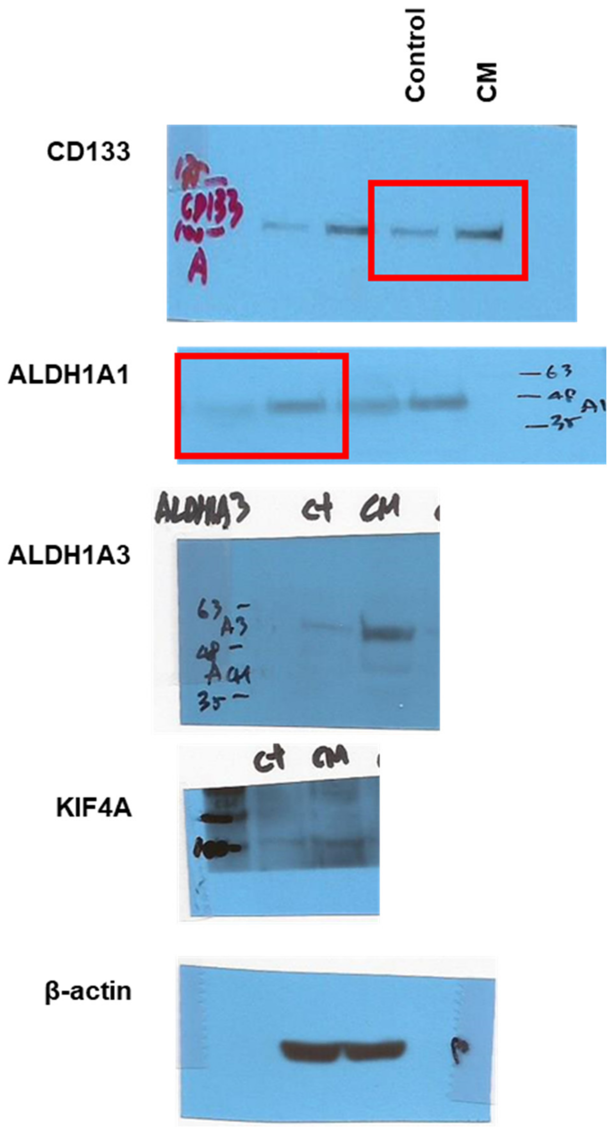

Figure S4: Uncropped Western Blot images for Figure 1A.

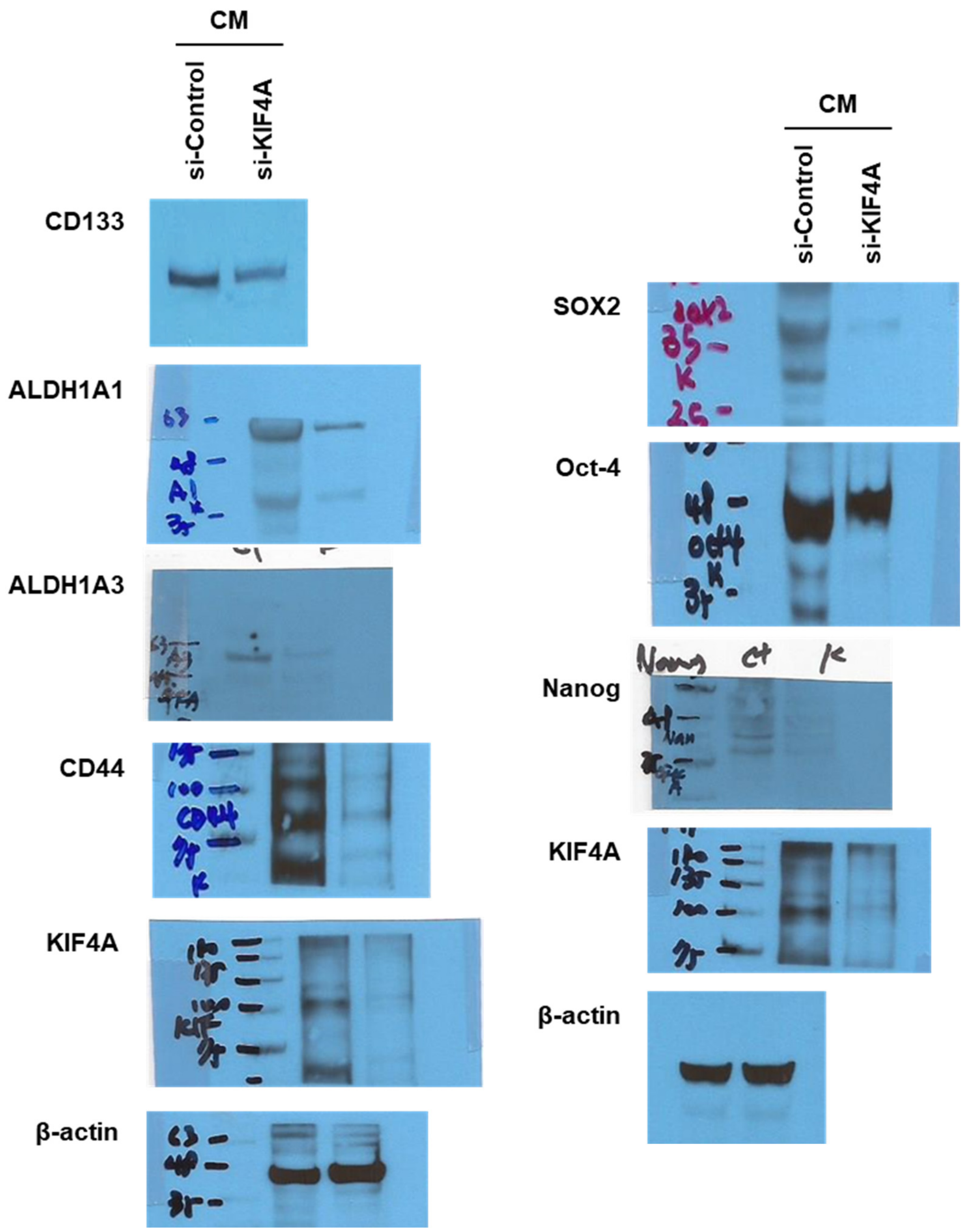

(**a**) (**b**)

Figure S5: (a) Uncropped Western Blot images for Figure 2D; (b) Uncropped Western Blot images for Figure 2E.

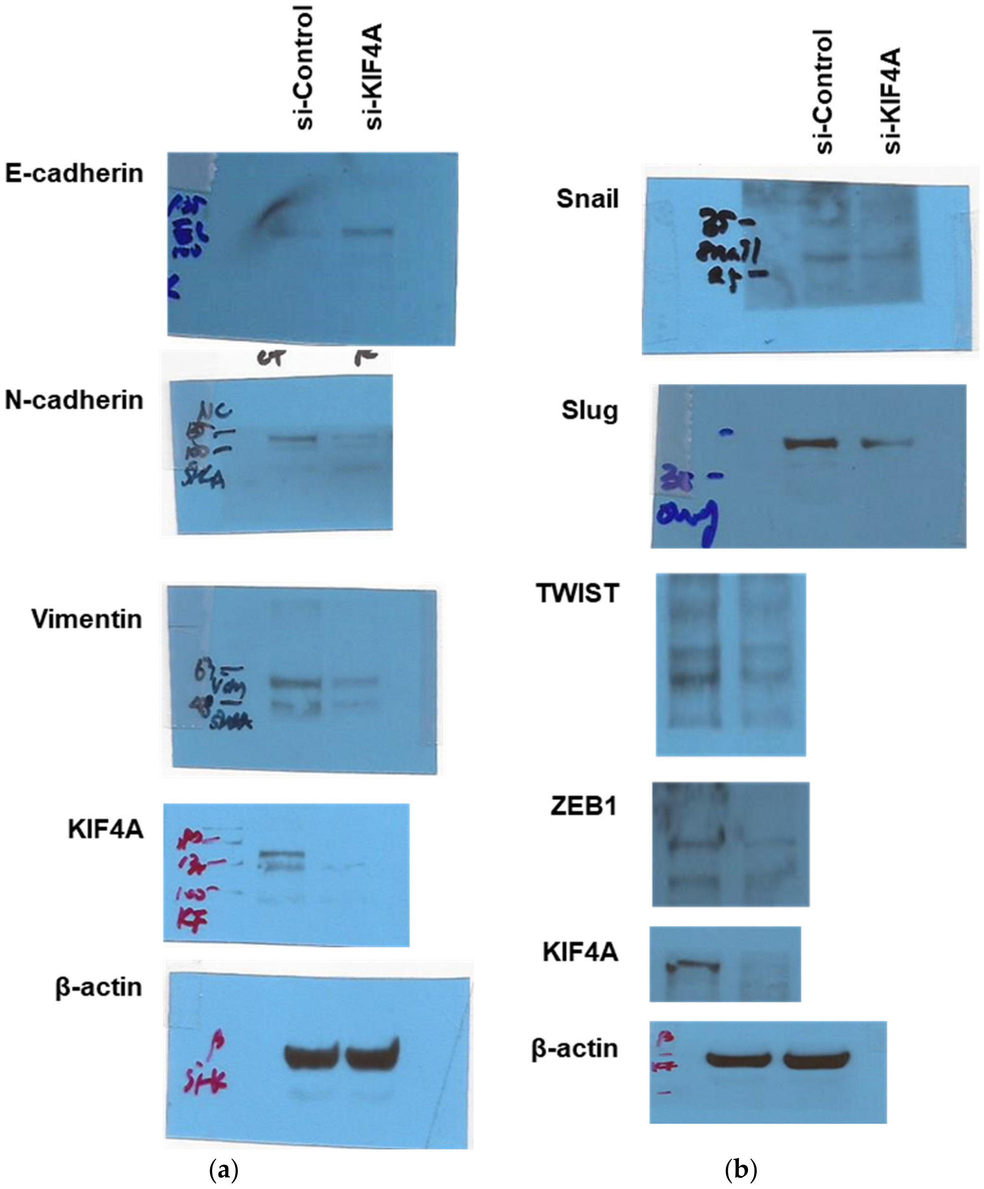

Figure S6: (a) Uncropped Western Blot images for Figure 3A; (b) Uncropped Western Blot images for Figure 3B.

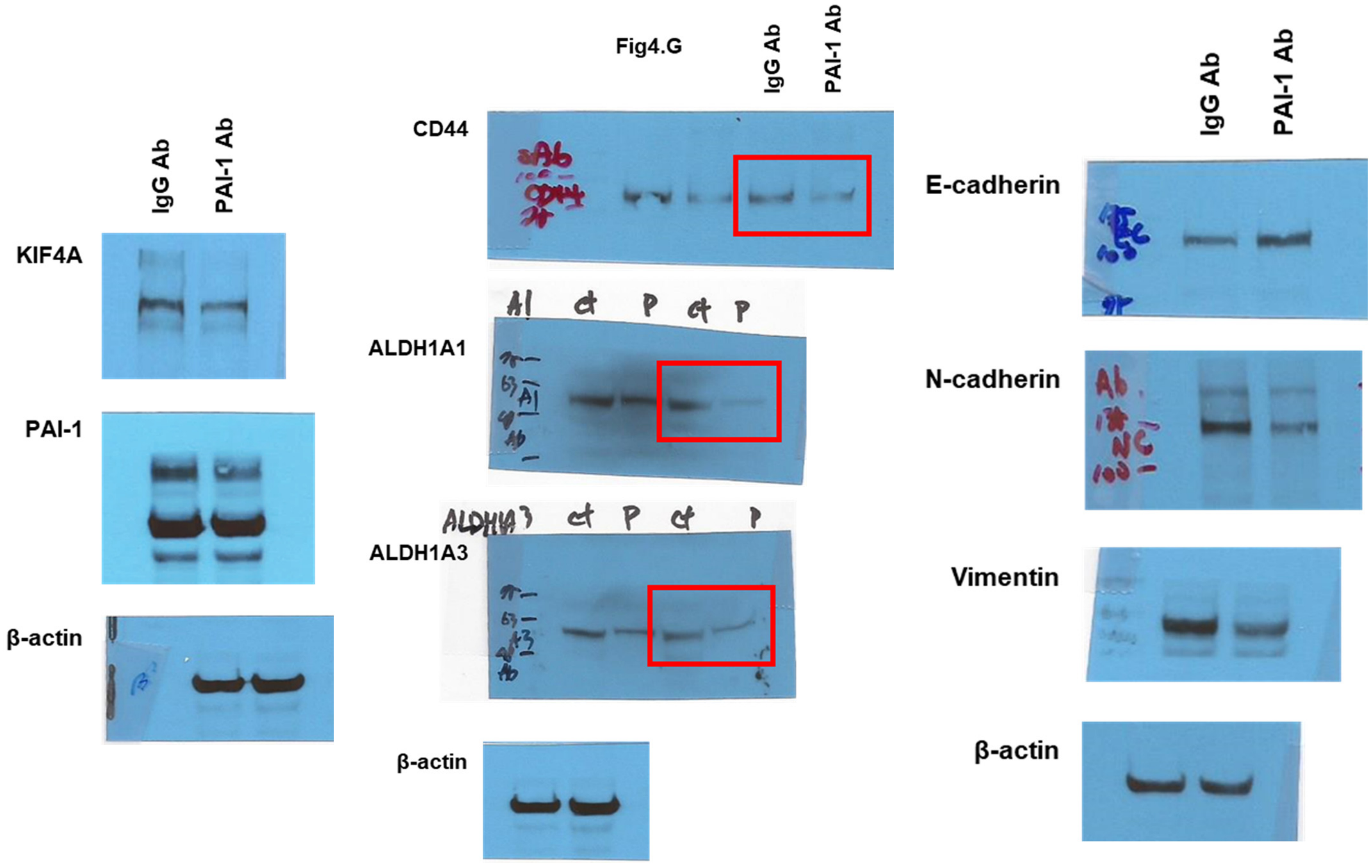

(a) (b) (c)

Figure S7: (a) Uncropped Western Blot images for Figure 4E; (b) Uncropped Western Blot images for Figure 4G; (c) Uncropped Western Blot images for Figure 4H.

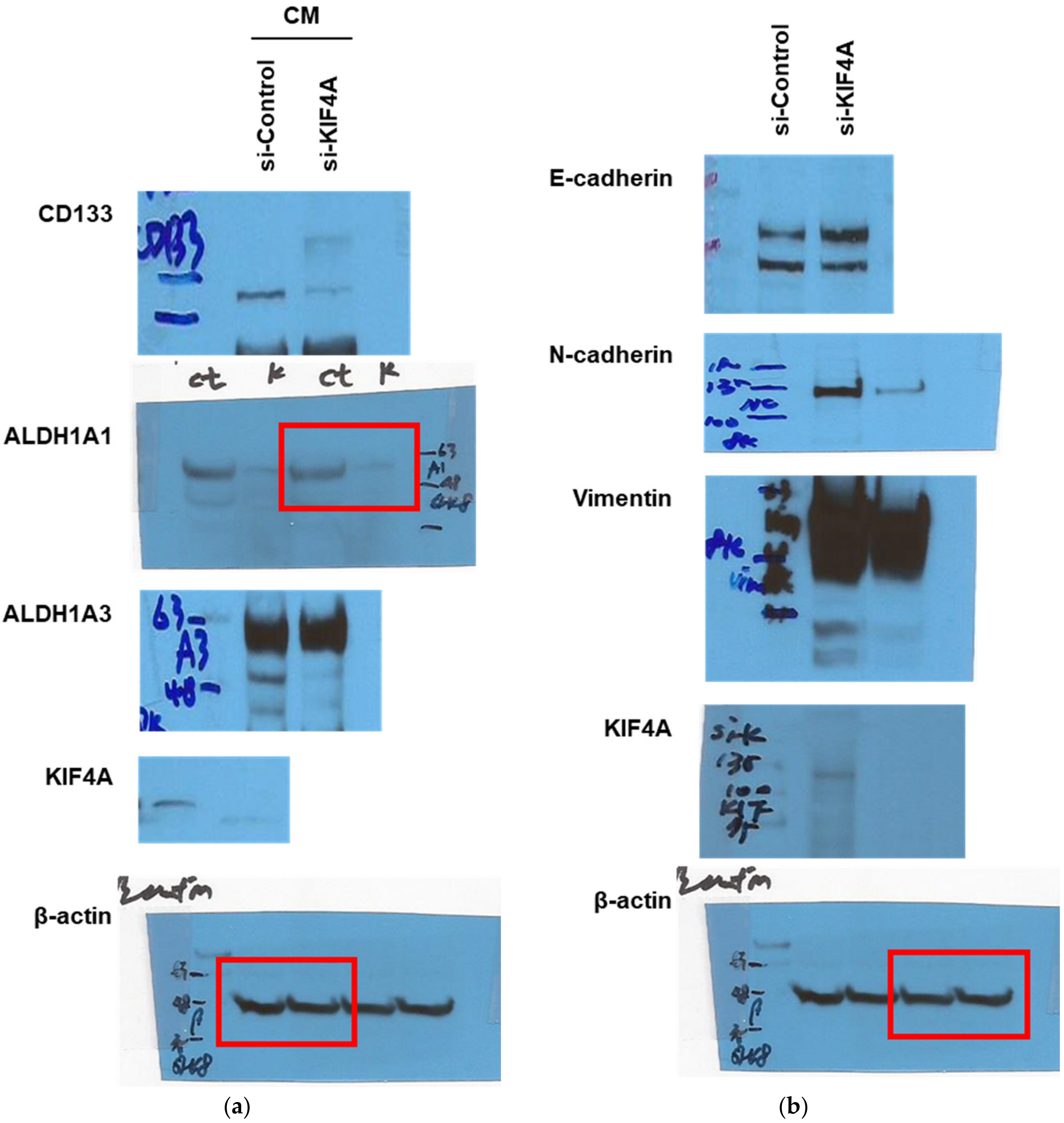

Figure S8: (a) Uncropped Western Blot images for Figure 5A; (b) Uncropped Western Blot images for Figure 5D.

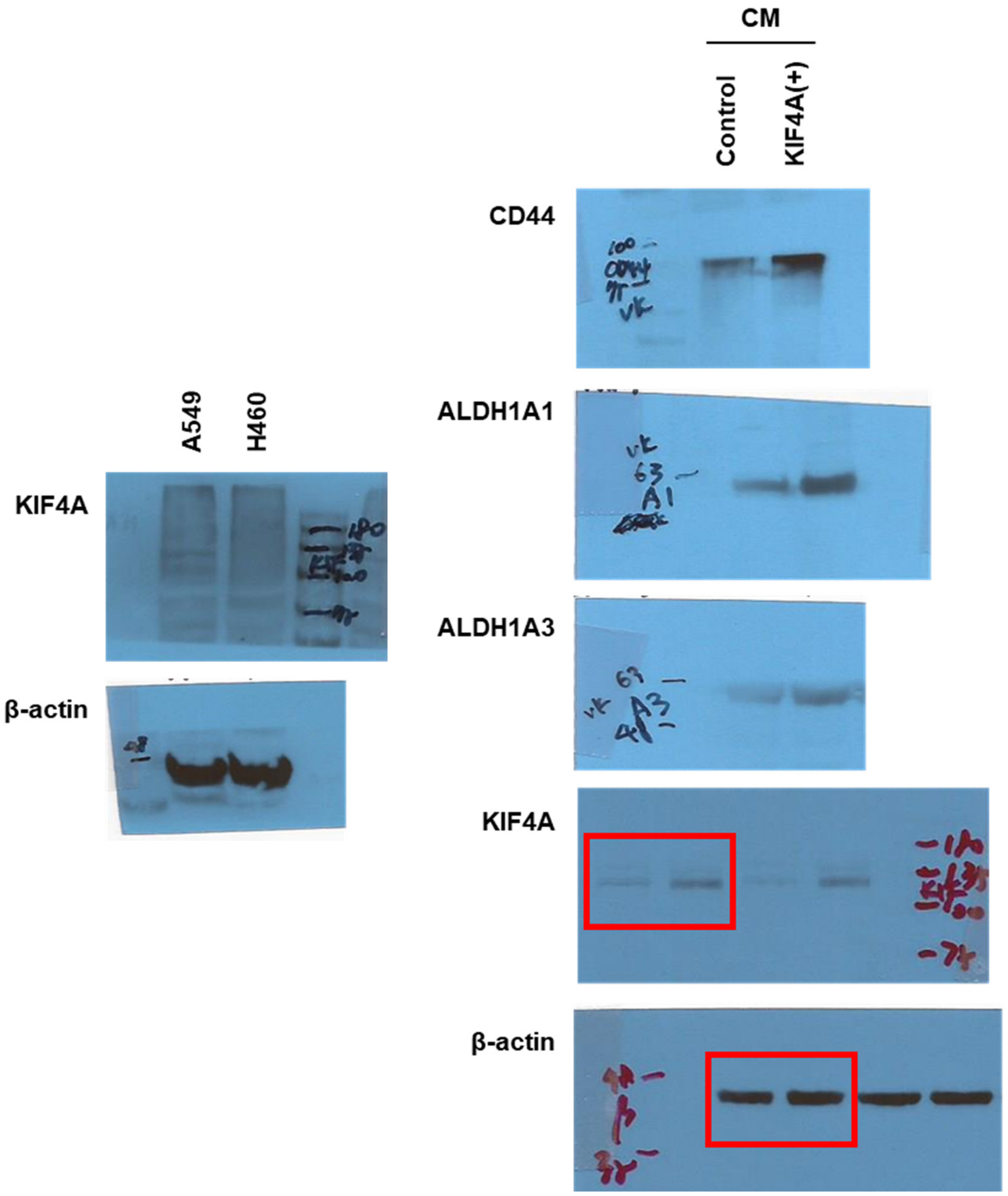

(**a**) (**b**)

Figure S9: (a) Uncropped Western Blot images for Figure S1A; (b) Uncropped Western Blot images for Figure S1B.

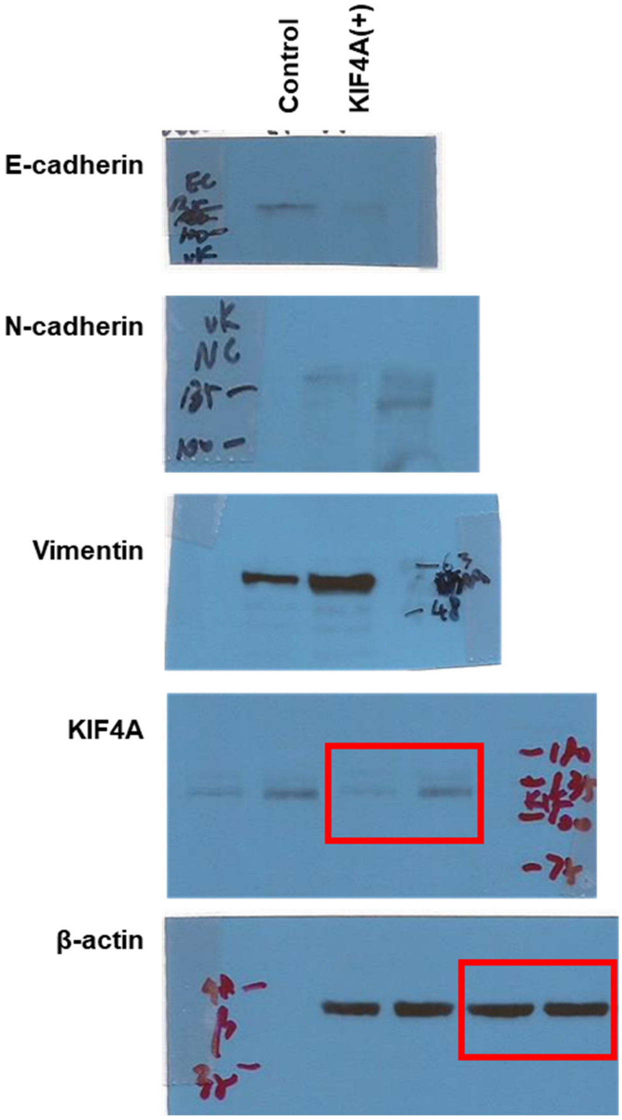

Figure S10: Uncropped Western Blot images for Figure S2B.

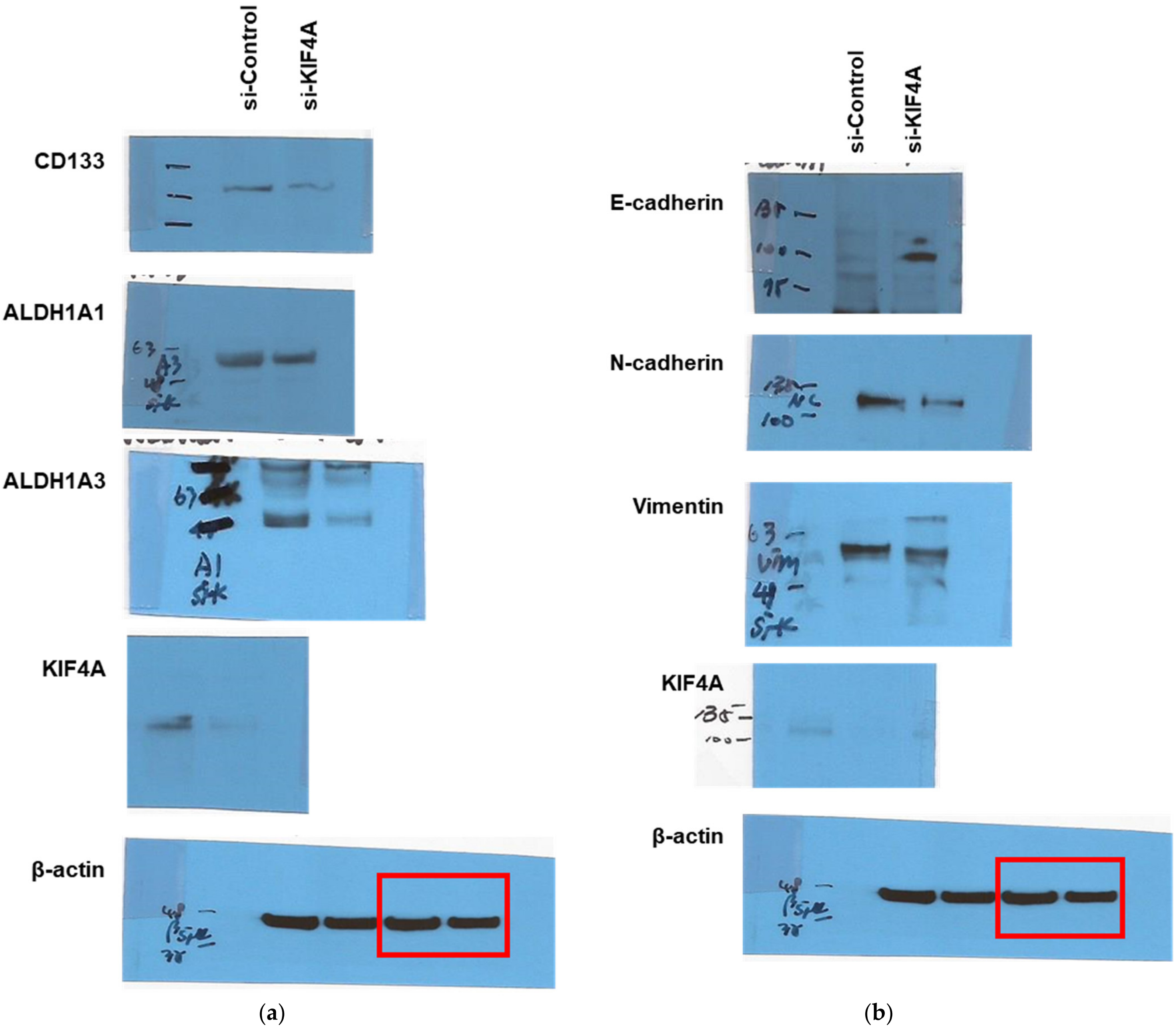

Figure S11: (a) Uncropped Western Blot images for Figure S3A; (b) Uncropped Western Blot images for Figure S3C.
